# Special Issue: Gene Therapy with Emphasis on RNA Interference

**DOI:** 10.3390/v7082830

**Published:** 2015-08-06

**Authors:** Kenneth Lundstrom

**Affiliations:** PanTherapeutics, Rue des Remparts 4, CH1095 Lutry, Switzerland; E-Mail: lundstromkenneth@gmail.com; Tel.: +41-79-776-6351

**Keywords:** gene therapy, non-viral delivery, viral vectors, RNA interference

## Abstract

Gene therapy was originally thought to cover replacement of malfunctioning genes in treatment of various diseases. Today, the field has been expanded to application of viral and non-viral vectors for delivery of recombinant proteins for the compensation of missing or insufficient proteins, anti-cancer genes and proteins for destruction of tumor cells, immunostimulatory genes and proteins for stimulation of the host defense system against viral agents and tumors. Recently, the importance of RNA interference and its application in gene therapy has become an attractive alternative for drug development.

## 1. Introduction

Since the late 1990s, gene therapy has been considered as one of the most promising approaches in modern medicine. However, setbacks occurring in treatment of a non-life-threatening disease by adenovirus [1] and children with severe combined immunodeficiency [2] with integration-competent retrovirus vectors caused some concern about the feasibility of gene therapy and resulted in decreased funding and reduced interest from the biotechnology and pharmaceutical industry. However, the positive outcome of the setbacks was a more systematic approach to the engineering of novel and safer gene therapy vectors and a strong emphasis in safety aspects of clinical trials. This has recently seen a rejuvenation of the field with more than 1800 gene therapy trials in progress or completed [3].

Gene therapy has commonly been described as the replacement of malfunctioning genetic information, substitution of heterologous genetic material or inhibition of harmful genetic products. In general, both non-viral and viral delivery systems have been developed for a large spectrum of indications such as cancer, hemophilia, muscular dystrophy, ophthalmological diseases and immunodeficiency. More recently, oligonucleotides and various RNA molecules based on RNA interference (RNAi) have proven their potential as reagents for gene therapy applications.

The Special Issue on Gene Therapy reflects the current applications of viral vectors with a special emphasis on RNAi. After the initial demonstration of the RNAi phenomenon, it took almost a decade to discover the important role of micro-RNA (miRNA) in gene regulation [4]. More than 600 human miRNA sequences have been discovered, many of which have been associated with human disease due to their involvement in transcription, signal transduction, cell cycle regulation, cell proliferation and growth, apoptosis, fat metabolism and neurogenesis [5]. A number of miRNAs have therefore been linked to cancer, cardiovascular, metabolic, infectious and neurological diseases among others. One advantageous feature of miRNAs is their reversible nature, which will allow therapeutic intervention without any permanent presence of drug after treatment termination. It also relates to epigenetic modifications originally demonstrated to be caused by aberrant DNA methylation or histone modifications, but more recently by changes in miRNA expression, which means that the genetic information remains intact. In this special issue, different delivery systems have been applied for RNAi-based gene therapy. Related to virology, there are two main applications of gene expressioninhibition. One is to target viral mRNA sequences to reduce or prevent virus replication. The other is to use viral vectors for targeting tumor promoting gene expression. As presented below, one article describes the effect of ribozyme-delivery to target human cytomegalovirus (hCMV) replication. In the three other papers, different viral vectors were employed for cancer therapy targeting specific genes related to tumor development.

## 2. Ribozyme-Based Approach

In attempts to target the overlapping mRNA region of the capsid assembly protein (AP) and protease (PR) essential for the replication of human cytomegalovirus (hCMV) an RNase P-based ribozyme variant was engineered by Yang and coworkers. In comparison to wild-type, the V718-A mutant ribozyme cleaved the AP mRNA target sequence efficiently showing a 60-fold higher activity *in vitro*. This resulted in 99% reduction of AP/PR expression, which significantly inhibited CMV growth in host cells. A closer examination suggested that the viral DNA encapsidation was substantially reduced blocking capsid assembly. The study demonstrated that ribozyme delivery is a potentially promising approach for anti-viral therapy.

## 3. Baculovirus-Mediated RNAi

Baculoviruses have been commonly used for gene transfer and recombinant protein expression within the field of biotechnology. Several studies have confirmed efficient gene delivery by baculoviruses in animal models [6]. Not long ago, baculovirus vectors were demonstrated to provide high potential for delivery of RNAi. In this context, Makkonen and co-authors review applications of baculovirus-based RNAi. For example, prevention of AcMNPV infection was obtained by RNAi delivery [7] and recombinant protein production was increased in *Trichoplusia ni* by an RNAi-approach [8]. Furthermore, as an example of an RNAi-based application in mammalian cells, baculovirus U6-driven short hairpin RNA (shRNA) efficiently decreased lamin A/C mRNA and protein levels in liver cell lines and in primate human hepatic stellate cells [9]. Interestingly, shRNAs demonstrated 95% suppression of gene expression in cultured rat glioma C6 cells and even 82% *in vivo* in rat brain [10]. Related to cancer therapy, baculovirus-based delivery of long non-coding RNA (lncRNA) generated inhibition of cell proliferation in hepatocelllular carcinoma cells (HCC) and in mice with HCC tumor implants [11]. Moreover, inhibition of miRNA-10b diminished the invasiveness of angiogenicity and tumor growth in a human glioma mouse model [12]. Baculoviruses have further been applied for RNAi approaches to inhibit viral replication, For instance, delivery of shRNA targeting the highly conserved core region of the hepatitis C virus (HCV) genome showed inhibition of viral replication in NNC#2 cells [13].

## 4. Alphaviruses and RNA Interference

As reviewed by Lundstrom, alphaviruses have frequently been used for recombinant protein expression in mammalian cells lines, primary cells and *in vivo* [14]. Moreover, alphavirus vectors in the form of replicon RNA, DNA/RNA layered vectors and recombinant viral particles have been subjected to immunization in animal models, which has provided protection against challenges with lethal doses of viral pathogens and tumor cells [15]. Furthermore, intratumoral and systemic delivery of alphavirus vectors has showed significant tumor regression and prolonged survival in animal tumor models. Among RNAi approaches, Semliki Forest virus (SFV)-based expression of neuron-specific miR124 sequences resulted in attenuated spread in the CNS and a significant improvement in survival of BLAB/c mice [16]. Due to the efficient RNA delivery and the extreme RNA replication, SFV vectors are considered as attractive candidates for RNAi-based gene therapy.

## 5. Lentivirus and Short Hairpin RNAs

Zhao and co-workers demonstrated the potential of lentivirus-based silencing of claudin 1 (CLDN1), a member of the tetraspan transmembrane protein family associated with tumor metastasis. This is the first demonstration of shRNA delivered by lentiviruses resulting in down-regulation of CLDN1 expression in MDA-MB-231 and MCF7 breast tumor cell lines. Furthermore, the CLDN1 knockdown led to reduced cell proliferation, cell survival and migration. Additionally, epithelial to mesenchymal transition (EMT) was inhibited as indicated by up-regulation of E-cadherin and down-regulation of smooth muscle cell α-actin (SMA) and Snai2. Although additional studies are necessary in other tumor cell lines and *in vivo*, the lentivirus approach has great potential for breast cancer therapy. 

## 6. Conclusions and Future Aspects

In addition to the examples described in this special issue on utilization of RNAi in gene therapy, additional studies have demonstrated the feasibility in lentivirus-based treatment of HIV [17]. For instance, lentivirus with an engineered polycistronic platform derived from the endogenous MVM7 gene was applied for the expression of a set of small antiviral RNAs, which was sufficient to provide protection against R5-tropic HIV in macrophages and reduced hematopoietic toxicity [18]. Moreover, survivin shRNA was evaluated for down-regulation of survivin expression for lentiviral-based delivery in the gastric cancer cell lines SGC-7901, MGC-803 and MKN-28 [19]. The outcome was significant decrease in survivin mRNA and protein levels and a significant inhibition of proliferation and migration of gastric cancer cells and tumorigenicity in mice with implanted tumors. Application of RNAi gene silencing was demonstrated for each of the three siRNAs (siE1A_4, siIVa2_2 and Pol-si2), which resulted in adenovirus inhibition in a dose dependent manner [20]. Combination of siRNAs did not increase adenovirus inhibition. However, combination therapy with the adenovirus receptor trap sCAR-Fc, the anti-viral drug cidofovir and the siRNAs, significantly increased the suppression of adenovirus replication.

Adeno-associated viruses (AAV) have been employed for therapeutic applications of RNAi delivery for myotonic dystrophy type 1 (DM1) [21]. AAV vectors carrying miRNA-based RNAi hairpins targeting the CUG^exp^ mRNA were intravenously administered in a human α-skeletal muscle actin long-repeat (HSA^LR^) mouse model of DMI. Delivery of AAV vectors significantly reduced disease pathology in muscles of injected animals. The indication of reversal of DMI by AAV-based miRNA delivery suggests the potential for long-term therapy. In another approach, AAV vectors were used for shRNA delivery to knock down the expression of Ac45 gene expression, which impaired osteoclast-mediated extracellular acidification and bone resorption *in vitro* and *in vivo* [22]. Furthermore, mice were protected against bone erosion and attenuated inflammation after local administration of Ac-45 shRNA by AAV.

The studies described above are just examples of the potential of applying RNAi in gene therapy. Anyway, it should have become clear to the reader that in relation to virology, delivery of shRNA and miRNA molecules is an attractive approach for targeting the inhibition of viral replication and thereby the spread of viral infections. Particularly attractive has been RNAi-based therapy of HIV patients. As important, is the approach to use various viral vectors for RNAi delivery. In this context, different diseases such as cancer and muscular dystrophy have been targeted demonstrating therapeutic efficacy in animal models. The approach of using RNAi has become especially attractive because of its reversible nature, which allows return to pre-treatment conditions after achieving therapeutic efficacy.

However, although efficacy in disease treatment has been achieved much work and development is still ahead. RNAi delivery poses still a serious problem and much attention is required for improved stability of RNAi molecules delivered by non-viral vectors. Similarly, efforts are needed for improvement of delivery by viral vectors. Essential questions to be addressed relate to control of gene expression and safety issues, especially of viral vectors. Furthermore, efforts are needed to develop technologies for more efficient and cost effective production of GMP grade material and to facilitate further clinical trials. It is expected that significant progress will provide the framework for making gene therapy the medicine of the future.
